# Dynetic-35 cobalt chromium balloon-expandable stent for iliac lesions: 12-month results of the BIONETIC-I multi-center study

**DOI:** 10.1186/s42155-025-00633-z

**Published:** 2025-12-13

**Authors:** Marianne Brodmann, Balázs Nemes, Nathalie Moreels, Martin Austermann, Jörg Schmehl, Jorn Robijn, Christos Rammos, Stefan Müller-Hülsbeck, Koen Keirse, Raphael Coscas, Karlis Kupcs, Anne Marie Augustin, Sven Moebius-Winkler, Michael Lichtenberg, Wouter Lansink

**Affiliations:** 1 Klinische Abteilung Für Angiologie, Medizinische Universität Graz, Graz, Austria; 2 Semmelweis University, Budapest, Hungary; 3 UZ Ghent—Universitair Ziekenhuis Gent, Ghent, Belgium; 4 St. Franziskus-Hospital GmbH, Gefäßchirurgie, Münster, Germany; 5 Universitätsklinikum Tübingen, Radiologische Universitätsklinik, Tübingen, Germany; 6 AZ Jan Portaels, Vilvoorde, Belgium; 7 Department of Cardiology and Vascular Medicine, West German Heart and Vascular Centre, University of Duisburg-Essen, Essen, Germany; 8 DIAKO Krankenhaus GmbH, Flensburg, Germany; 9 Regionaal Ziekenhuis Heilig Hart Tienen, Tienen, Belgium; 10 Hopital Ambroise Paré, Boulogne Billancourt, France; 11 Pauls Stradins Clinical University Hospital, Riga, Latvia; 12 Klinikum Bayreuth, Bayreuth, Germany; 13 Universitätsklinikum Jena, Jena, Germany; 14 Karolinen Hospital Huesten, Arnsberg, Germany; 15 Ziekenhuis Oost-Limburg, Genk, Belgium

**Keywords:** Peripheral artery disease (PAD), Iliac artery stenting, Cobalt chromium stent, Endovascular treatment, Atherosclerotic lesions

## Abstract

**Purpose:**

The BIONETIC-I trial aimed to evaluate the safety and effectiveness of the cobalt chromium Dynetic-35 stent, used in conjunction with the Passeo-35 Xeo peripheral dilation catheter, for treating atherosclerotic lesions in iliac arteries of patients with peripheral artery disease (PAD).

**Materials and methods:**

This international, multi-center, prospective, single-arm study enrolled 160 subjects with 212 lesions across six European countries. The primary endpoint was a composite of major adverse events (MAE) at 12 months, including device- or procedure-related death within 30 days, clinically driven target lesion revascularization (cdTLR), and major index limb amputation. Secondary endpoints included technical and procedural success, cdTLR rate, mortality, major amputation rate, primary patency, and changes in PAD-related parameters.

**Results:**

The patients (61.9% male, median age 65 years) presented with predominantly calcified lesions (90.1%) and high-grade stenosis (average 85.5%), with 12.5% having CLTI. The 12-month MAE rate was 3.5% (97.5% upper confidence limit: 7.2%), significantly non-inferior to the pre-specified performance goal (*p* < 0.0001). Secondary endpoints showed favorable 12-month outcomes, including a low cdTLR rate (2.0%), robust core lab-reported primary patency (93.1%), and significant improvements in PAD-related parameters such as Ankle-Brachial Index, Rutherford classification, and Walking Impairment Questionnaire. Subgroup analysis revealed no differences in MAEs between patients with severe/moderate and mild/no calcification, with notable improvements in functional measures for those with severe/moderate calcification.

**Conclusion:**

The balloon-expandable cobalt chromium stent Dynetic-35 demonstrated safety and effectiveness in treating iliac arteries at the 12-month timepoint, showing promising results across various patient subgroups, including those with calcified lesions.

**Level of evidence:**

Level 2, therapeutic study.

**Trial registration:**

ClinicalTrials.gov, NCT04830228. Registered 31 March 2021, https://clinicaltrials.gov/ct2/show/NCT04830228.

## Introduction

Peripheral artery disease (PAD) affecting the iliac artery reduces blood flow to the lower extremities, causing symptoms like leg cramping and weakness, particularly during physical activity [1]. Complications can include amputation, increased heart disease risk, and mortality [2, 3]. PAD is caused by atherosclerotic lesions [4]. While open surgery was traditionally the primary treatment, less invasive endovascular procedures are now preferred for treating iliac artery atherosclerotic lesions [5]. These minimally invasive techniques offer reduced risks and faster recovery times [6]. Several studies indicate a preference for iliac stenting over percutaneous transluminal angioplasty (PTA) [7, 8].

Balloon-expandable cobalt chromium stents are particularly suitable for the iliac artery due to their radial force, precise placement, and flexibility [9, 10]. Historical studies have demonstrated the efficacy and safety of balloon-expandable stents (BES) in treating iliac artery conditions [11]. Previous trials like VISIBILITY, MELODIE, ACTIVE, and SENS-ILIAC have further investigated BES, showing promising safety and efficacy profiles with low major adverse event rates and high primary patency rates [12–15]. However, there is currently a lack of prospective clinical evidence regarding the safety and efficacy of cobalt chromium stents in individuals with atherosclerotic disease affecting the iliac arteries. This study aimed to further assess the safety and efficacy of the Dynetic-35 balloon-expandable stent (BIOTRONIK, now Teleflex) in real-world use within its CE-approved indication.

## Materials and methods

### Patient selection and study design

BIONETIC-I is an international, multi-center, prospective, non-inferiority, single-arm clinical study designed to evaluate the safety and effectiveness of Dynetic-35 balloon-expandable stent for the treatment of peripheral artery disease (PAD) in subjects with atherosclerotic lesion(s) of the iliac arteries. The study included 160 subjects from 15 clinical trial sites across 6 countries: Austria, Belgium, France, Germany, Hungary, and Latvia. Follow-up is scheduled at 1, 2, and 5 years post-procedure. We report here the 12-month results. Longer-term follow-up results will be reported in a subsequent publication.

An independent Clinical Events Committee (CEC) was responsible for adjudication of endpoint-related adverse events. The core laboratory Black Forest GmbH (Bad-Krozingen, Germany) independently review ultrasound and angiography analyses. The trial was registered on the National Institutes of Health website (ClinicalTrials.gov; identifier NCT04830228).

Eligible subjects were adults (≥ 18 years) with PAD, presenting with de novo, restenotic (excluding in-stent restenosis), or occluded atherosclerotic lesions in the iliac arteries. Key inclusion criteria included Rutherford category 2 or higher, ≥ 70% stenosis in the iliac artery, and reference vessel diameter between 5 and 10 mm. Major exclusion criteria encompassed in-stent lesions, pre-existing target iliac artery aneurysm, perforation or dissection, and abdominal aortic aneurysm contiguous to the target iliac artery.

### Study device

The primary investigational device was the Dynetic-35 stent system, a balloon-expandable cobalt chromium stent designed for peripheral iliac lesions. Compared to the earlier Dynamic stent, Dynetic-35 incorporates cobalt chromium and thinner struts for improved strength and flexibility. The stent was available in diameters ranging from 5 to 10 mm and lengths from 18 to 78 mm. The delivery system was compatible with a 6F sheath and featured an over-the-wire (OTW) design with a 0.035″ guidewire lumen, available in usable lengths of 90, 130, and 170 cm. The Passeo-35 Xeo peripheral dilation catheter is an OTW percutaneous transluminal angioplasty (PTA) dilation catheter. It is intended for stenosis dilation in the peripheral vessels and the arteriovenous dialysis fistulae. Passeo-35 Xeo was used for the pre-dilation (when performed) of the atherosclerotic iliac artery before the placement of Dynetic-35 stent and/or for the post-dilation (when performed) after the placement of Dynetic-35 stent.

### Study outcome measures

The primary endpoint was the major adverse events (MAE) rate at 12 months, a composite of device- or procedure-related death within 30 days post-procedure, clinically driven target lesion revascularization (cdTLR), and major index limb amputation. The study was designed to test non-inferiority against a predefined performance goal.

Secondary endpoints included technical success, procedural success, rates of cdTLR, mortality, major index limb amputation, and primary patency. Technical success was defined as the successful delivery and deployment of the Dynetic-35 stent with residual stenosis ≤ 30% as determined by angiography. Procedural success was defined as technical success combined with no MAEs from enrollment through discharge. Primary patency was defined as peak systolic velocity ratio < 2.5 by duplex ultrasound and absence of cdTLR. Changes in Rutherford classification, Ankle-Brachial Index (ABI), and Walking Impairment Questionnaire (WIQ) scores were also assessed at follow-up time points.

### Statistical analysis

For qualitative variables, absolute and relative frequencies were determined, with exact binomial 95% confidence intervals for proportions when relevant. Survival analysis using the Kaplan–Meier estimator was performed for safety endpoints, including two-sided 95% confidence intervals and survival curves. The primary endpoint (MAE rate) was compared to the hypothesized rate of 14% based on [16], using an exact one-sided binomial test for non-inferiority. For paired data comparisons between baseline and follow-up, Student’s *t*-test or Wilcoxon signed-rank test was used for quantitative variables, depending on the normality of distribution. For qualitative ordinal variables, the sign test or Wilcoxon signed-rank test was employed. Statistical analysis was performed using SAS version 9.4 (SAS Institute Inc., Cary, NC, USA) and R version 4.3.2 (R Foundation for Statistical Computing, Vienna, Austria).

## Results

### Patient characteristics

Among the 160 participants enrolled between August 2021 and January 2023, 61.9% were male, with a median age of 65.0 years and a median BMI of 25.7 kg/m^2^ (Table 1). Current smokers accounted for 51.9% of the cohort, while a significant proportion had hypertension (77.5%) and hyperlipidemia (74.4%). History of PAD was reported in one third (33.1%) of the participants, with smaller percentages indicating diabetes (21.9%), coronary artery disease (31.9%), renal disease (5.6%), or cancer (14.4%). In terms of PAD-related function, mean Ankle-Brachial Index (ABI) was 0.71 ± 0.2 and 54.4% were Rutherford 3, with 12.5% of the subjects having a chronic limb-threatening ischemia (Rutherford 4, 5, 6). Finally, the mean overall WIQ score was low (32.4 ± 22.3).

**Table 1:** Baseline characteristics

	*N*	%
Gender	160	
Male	99	61.9
Age at time of enrollment [years]		
* N*	160	
Median (Q1–Q3)	65.0 (57.5–71.5)	
BMI [kg/m^2^]		
* N*	160	
Median (Q1–Q3)	25.74 (23.41–28.25)	
Coronary artery disease	51	31.9
Cerebrovascular disease	20	12.5
History of PAD	53	33.1
Hypertension	124	77.5
Hyperlipidemia	119	74.4
Renal disease	9	5.6
Yes, no dialysis	8	5.0
Yes, under dialysis	1	0.6
Diabetes	35	21.9
Insulin dependent	16	10.0
Non-insulin dependent	19	11.9
Smoking history		
Never smoked	14	8.8
Ex-smoker	63	39.4
Current smoker	83	51.9
Cancer	23	14.4
Rutherford classification		
Category 2	53	33.1
Category 3	87	54.4
Category 4	15	9.4
Category 5	4	2.5
Category 6	1	0.6
CLTI—Rutherford classification (4, 5 or 6)	20	12.5
ABI of target limb(s)		
* N*	190	
Mean ± SD	0.714 ± 0.2165	
WIQ score overall		
* N*	152	
Mean ± SD	32.39 ± 22.254	
Impaired kidney function (eGFR below 60 mL/min/1.73 m^2^)	*N* = 122	
Yes	23	18.9

### Lesion characteristics

Overall, 212 lesions were treated with Dynetic-35, mostly in the common iliac artery (CIA) (79.2%) and external iliac artery (EIA) (19.3%). Mean lesion length was 30.7 mm (± 17.6 mm) (Table 2). 10.4% were occluded, with average occlusion length of 47.2 mm (± 21.9 mm). Pre-procedure stenosis averaged 85.5% (± 9.3%). 90.1% of lesions showed calcification based on visual estimation, 30.7% severe. TASC classification consisted of type A (60.8%), B (34.4%), C (3.8%), and D (0.9%). Average reference vessel diameter was 8.0 mm (± 1.1 mm).

**Table 2:** Lesion characteristics

	*N*	%
Access to target lesion	*N* = 212	
Sub-intimal	4	1.9
Intra-luminal	208	98.1
Lesion location	*N* = 212	
Common iliac artery (CIA)	168	79.2
External iliac artery (EIA)	41	19.3
Internal iliac artery (IIA)	0	0.0
CIA and EIA	3	1.4
CIA and IIA	0	0.0
Indication for treatment with Dynetic-35	*N* = 212	
De novo lesion	185	87.3
Occlusion	22	10.4
Re-stenosis	5	2.4
Re-occlusion	0	0.0
Calcification	*N* = 212	
None	21	9.9
Mild	59	27.8
Moderate	67	31.6
Severe	65	30.7
TASC classification	*N* = 212	
Type A	129	60.8
Type B	73	34.4
Type C	8	3.8
Type D	2	0.9
Thrombus present	4/212	1.9
Lesion length (visual estimate) [mm]		
* N*	212	
Mean ± SD	30.73 ± 17.552	
Reference vessel diameter (visual estimate) [mm]		
* N*	212	
Mean ± SD	8.00 ± 1.069	
Length of occlusion [mm]		
* N*	22	
Mean ± SD	47.20 ± 21.878	
Stenosis pre-procedure (visual estimate) [%]		
* N*	212	
Mean ± SD	85.49 ± 9.289	
Residual stenosis after Dynetic-35 implantation [%]		
* N*	212	
Mean ± SD	3.73 ± 9.49	
Multiple implanted Dynetic-35 stents were placed	*N* = 15	
Kissing stent	1	6.7
Long lesion	1	6.7
Overlapping	13	86.7

### Procedure characteristics

The mean procedure duration was 41.2 ± 26.4 min, with a wide range from 9 to 140 min (Table 3). Most patients (68.8%) had one target lesion treated, 30% had two lesions treated, and 1.2% had 3 lesions. The femoral artery was the predominant vascular access site (91%), followed by brachial (8.6%) and radial (0.5%). Hemostasis was primarily achieved using vascular closure devices (76.1%) or manual compression (20.6%), with a mean time to hemostasis of 5.9 ± 6.1 min. Mean Dynetic-35 length was 40.7 mm, and the mean diameter measured 8.05 mm. These values are consistent with the average reference vessel diameter of 8.0 mm and the average lesion length of 30.7 mm. This corresponds to the instructions for use, which recommend selecting a stent diameter that matches the vessel diameter, achieving a final stent-to-vessel ratio of 1:1, and ensuring full lesion coverage with slight extension into adjacent healthy vessel segments.

**Table 3:** Procedure characteristics

	*N*	%
Procedure duration [min]		
*N*	160	
Mean ± SD	41.24 ± 26.372	
Contrast medium amount [mL]		
*N*	160	
Mean ± SD	79.97 ± 46.195	
Number of target lesions treated (on subject level)	*N* = 160	
1	110	68.8
2	48	30.0
3	2	1.3
Mean ± SD	1.33 ± 0.496	
Number of introducer sheath(s) used during index procedure	*N* = 160	
1	110	68.8
2	50	31.3
Mean ± SD	1.31 ± 0.465	
Vascular access body side	*N* = 210	
Left	105	50.0
Right	105	50.0
Vascular access site	*N* = 210	
Femoral	191	91.0
Brachial	18	8.6
Radial	1	0.5
Popliteal	0	0.0
Pedal	0	0.0
Time cath lab/operating theater used [min]		
*N*	160	
Mean ± SD	58.96 ± 36.001	
Number of vascular access sites	*N* = 160	
0	0	0.0
1	111	69.4
2	49	30.6
Pre-dilatation	82/212	38.7
Post-dilatation	35/212	16.5
Vascular closure activity	*N* = 209	
Angio Seal 6Fr	1	0.5
Combination	3	1.4
Dedicated (mechanical) compression device	1	0.5
Manual compression	43	20.6
Surgical closure	2	1.0
Vascular closure device	159	76.1
Time to hemostasis [min]		
*N*	209	
Mean ± SD	5.88 ± 6.104	
Discharge post-procedure [days]		
*N*	160	
Mean ± SD	1.36 ± 1.854	

### Primary endpoint

In BIONETIC-I, the major adverse event (MAE) rate, a composite of device- or procedure-related death within 30 days post index procedure, clinically driven target lesion revascularization (cdTLR), and major index limb amputation up to 12 months post index procedure, was observed to be 3.5% (5 out of 144 patients, comprised of four cdTLRs and one major target limb amputation (in a CLTI patient)) at 12 months after the intervention. The upper 97.5% confidence limit for this rate was 7.2%. This MAE rate was significantly lower (*p* value < 0.0001) than the predefined non-inferiority threshold of 14%. In a sensitivity analysis with a most stringent approach, also considering events until the upper window of 12 months after procedure (395 days in contrast to up to 365 days) for patients who had 12-month visit (or later follow-up visit) or experienced the event, one additional event can be identified. This event occurred in a patient who had a follow-up visit at day 340 and a clinically driven target lesion revascularization (cdTLR) at day 374. With the inclusion of this additional event, the major adverse event (MAE) rate increased to 4.2%, with an upper confidence limit of 8.1%, remaining below the predefined non-inferiority threshold.

### Secondary endpoints

**Fig. 1:**
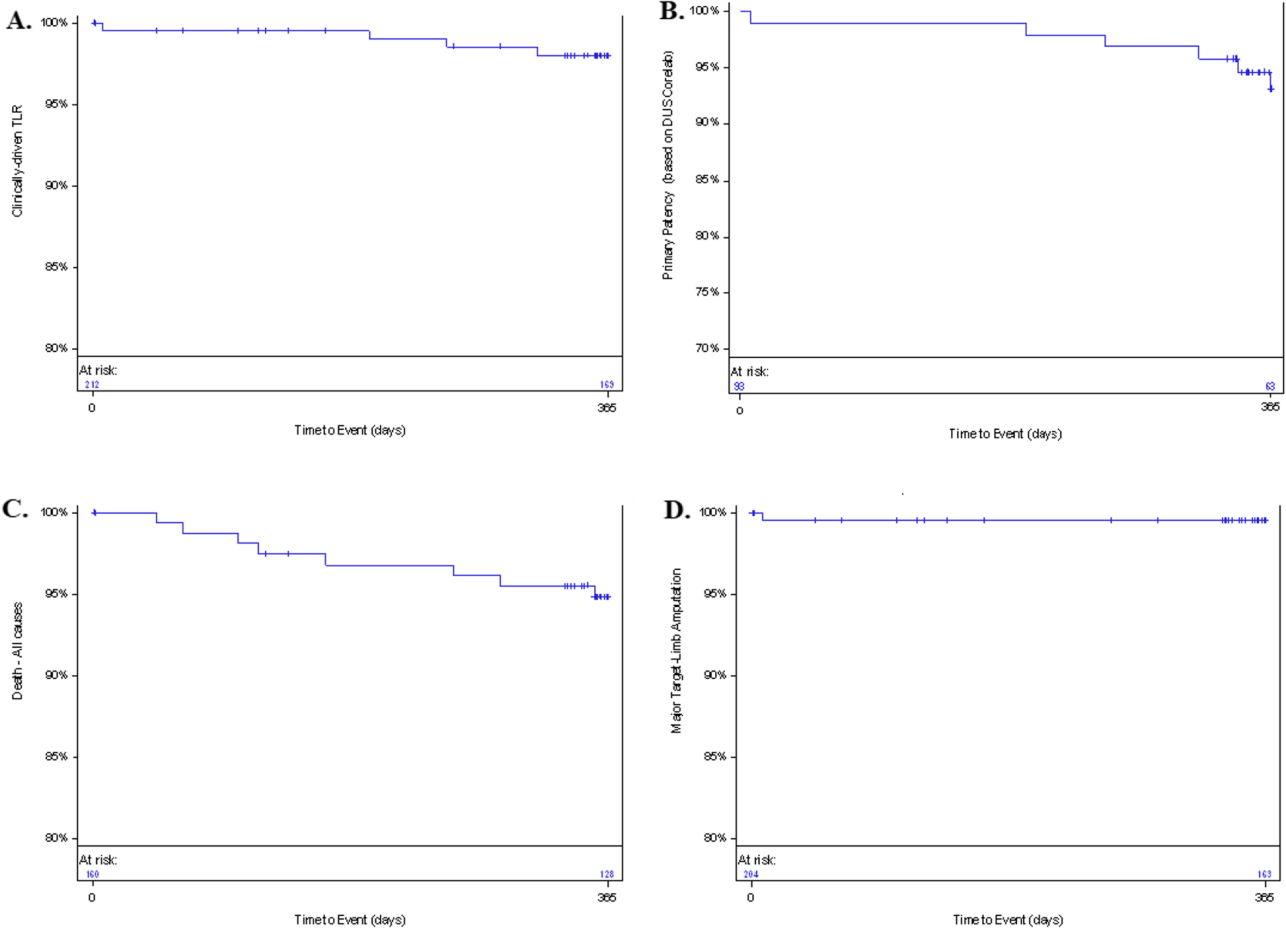
Secondary endpoints at 12 months. **A** Kaplan–Meier for cdTLR. **B** Kaplan–Meier for primary patency (core lab adjudicated). On lesion level, only lesions which had a valid assessment by the core lab are considered. **C** Kaplan–Meier for overall survival. **D** Kaplan–Meier for major target limb amputations

The BIONETIC-I study demonstrated favorable secondary endpoints for the Dynetic-35 stent. Technical success rates were high, with the Dynetic-35 stent achieving 98.6% at the lesion level (209/212 lesions) and 98.1% at the subject level (157/160 subjects). The Passeo-35 Xeo balloon showed 100% device success for both pre-dilation (57/57 devices) and post-dilation (17/17 devices). At 12 months, freedom from clinically driven target lesion revascularization was 98.0% (95% CI: 94.8%, 99.2%), with only four events occurring during the follow-up period (Fig. 1A). Primary patency rates were robust with core lab-assessed rates of 93.1% (95% CI: 85.1%, 96.9%) (Fig. 1B).

Regarding mortality, the Kaplan–Meier estimate was 5.2% (95% CI: 2.6%, 10.1%) at 365 days (Fig. 1C), with eight deaths reported during the follow-up period. Importantly, there was 99.5% (95% CI: 96.5%, 99.9%) freedom from major target limb amputation at 365 days (Fig. 1D). Only one major amputation event occurred, which took place 8 days post-procedure and was in a patient with Rutherford category 6 (major tissue loss) at baseline. The amputation was independently adjudicated as not related to the device nor the procedure.

**Fig. 2:**
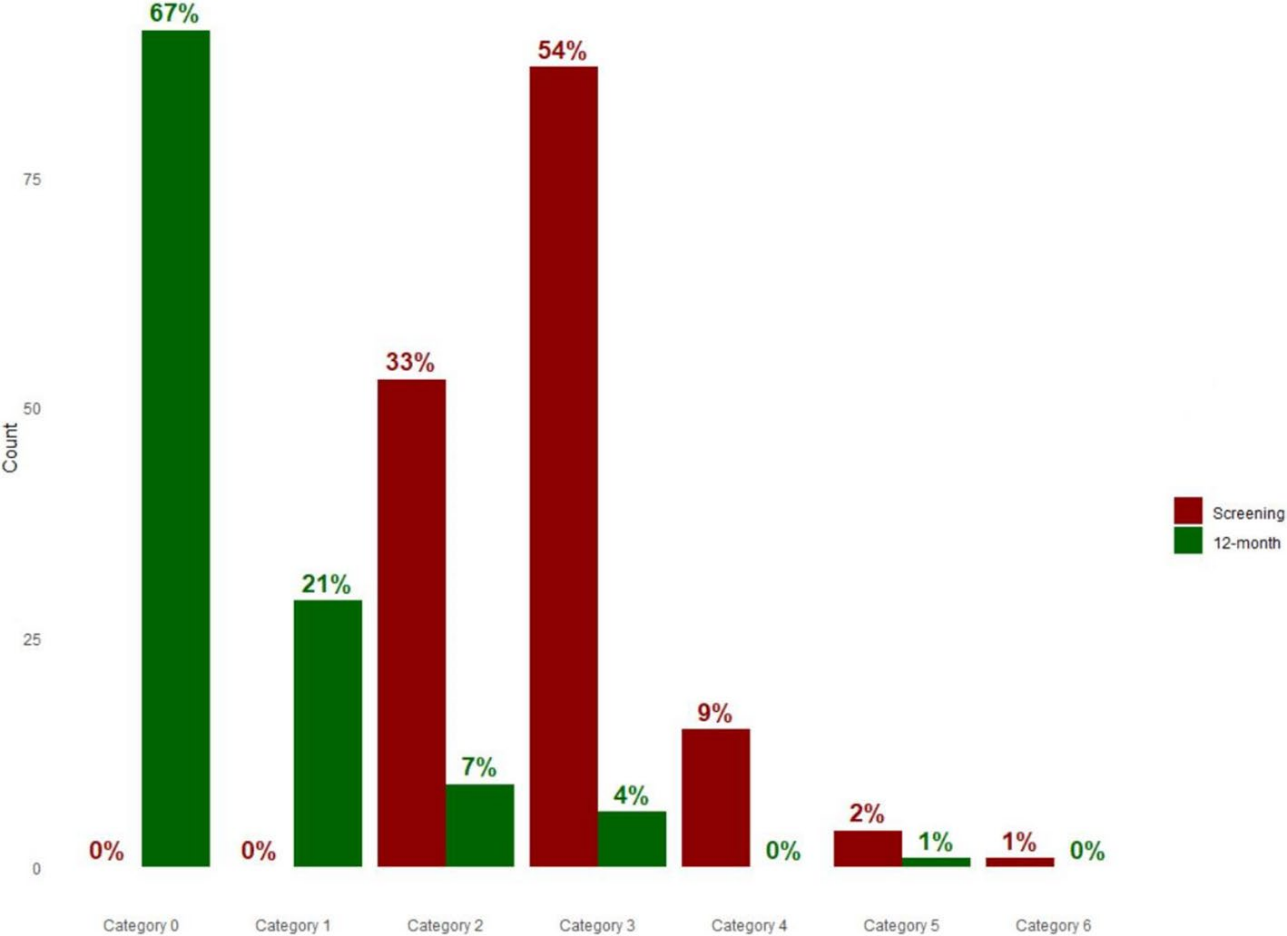
Change in Rutherford between screening and 12-month follow-up

All patient outcomes also improved significantly. At 12 months, 93.4% of participants improved by at least one Rutherford category (Fig. 2), with critical limb ischemia prevalence decreasing from 12.5% at baseline to 0.7% at 12 months. The mean Ankle-Brachial Index increased from 0.71 at screening to 0.93 at 12 months (*p* < 0.0001), indicating improved blood flow (Table 4). Additionally, the WIQ score rose from 32.4 at screening to 73.9 at 12 months (*p* < 0.0001), reflecting substantial improvement in patients’ walking ability (Table 4).

**Table 4:** Changes in ABI and WIQ from screening to 12-month follow-up

	Screening	12 months	Paired *p* value
ABI target limb	*N* = 190	*N* = 161	*N* = 155
Mean ± SD	0.71 ± 0.22	0.93 ± 0.21	< 0.0001
WIQ overall score	*N* = 152	*N* = 131	*N* = 125
Mean ± SD	32.4 ± 22.3	73.9 ± 30.1	< 0.0001

### Subgroup analyses

There was no difference in MAE rates at 365 days between the patients with severe/moderate calcification and those with mild/no calcification (3.1% vs. 3.6%, *p* value = 0.91). Importantly, even within the severe/moderate calcification subgroup, clinically and statistically significant improvements were observed at 12 months compared to baseline in several important functional measures, including target limb ABI, Rutherford classification, and total WIQ scores (Table 5).

**Table 5:** ABI, Rutherford, and WIQ by calcification subgroup

	Calcification subgroup			
	Mild/none		Severe/moderate	
Change in ABI target limb 12 months vs. screening (paired data)	*N* = 57	%	*N* = 98	%
Mean ± SD	0.251 ± 0.28		0.196 ± 0.23	
* p* value	< 0.0001		< 0.0001	
Improvement of ≥ 1 point in Rutherford at 12 months (paired data)	47/50	94.0	80/86	93.0
Change in WIQ overall score 12 months vs. screening (paired data)	*N* = 47		*N* = 78	
Mean ± SD	40.75 ± 30.3		40.54 ± 31.36	
* p* value	< 0.0001		< 0.0001	

## Discussion

The BIONETIC-I study evaluated the 12-month safety and efficacy of Dynetic-35 cobalt chromium stent and Passeo-35 Xeo peripheral dilation catheter for treating PAD in iliac arteries. While similar to other atherosclerotic iliac disease studies in terms of age, gender, comorbidities, and majority TASC A and B lesions, it distinguished itself by including a higher proportion of complex lesions based on calcification and preprocedural stenosis [17]. The mean preprocedural stenosis of 85.5% in BIONETIC-I was higher than other major iliac studies, some examining covered stents, such as 69.3% in iCARUS, 68.3% in VISIBILITY [12], and 62.9% in MELODIE [13]. Overall, despite challenging lesions, the technical success of Dynetic-35 was high, in-line with a previous single-center retrospective study of this stent [18].

The primary endpoint showed a relatively low major adverse events (MAE) rate of 3.5% at 12 months, which compares favorably to rates reported in similar studies. Torsello et al. [16] reported a 6.9% MAE rate for the Dynamic bare metal stent, while Krankenberg et al. [19] demonstrated a 7.3% rate in the balloon-expandable arm of the ICE trial. The MELODIE trial [13] exhibited an 8.9% MAE rate with a stainless-steel balloon-expandable stent. Studies on self-expandable iliac stents showed 12-month MAE rates ranging from 2.1% for the nitinol Astron stent [20] to 5.4% in the ORION trial [21].

Covered stents are typically reserved for more challenging anatomical and pathological lesions due to more favorable 12-month patency in complex lesions as suggested by the COBEST trial and subsequent meta-analysis comparing bare metal stent to balloon-expandable covered stent [22–24]. In addition, covered stent may decrease the risk of distal embolization and vessel rupture in heavily calcified iliac lesion [25]. In the BOLSTER study [26], while lesions presented moderate to severe calcification similar to BIONETIC-I, the LIFESTREAM balloon-expandable covered stent showed a 9-month MAE rate of 4.5%. The iCARUS trial which evaluated iCast/Advanta V12 covered stent demonstrated a 9-month primary composite endpoint rate of 8.1% [17] while the VBX Flex study reported a 9-month MAE rate of 2.3% [27]. Thus, despite a more occlusive disease, Dynetic-35 compares well with covered stents.

Secondary endpoints in BIONETIC-I were consistent with findings from previous iliac artery studies. The cdTLR rate of 2% closely paralleled the 2.6% rate observed in the BIODYNAMIC study [16]. The primary patency in BIONETIC-I was 93.1%, higher than the 89.8% rate reported for the Astron stent [20]. The major target limb amputation rate of 0.5% was similar to the 0.4% rate reported by Krankenberg et al. [19]. All-cause mortality was 5.2% at 12 months, lower than 6.9% for the cobalt-chromium BES arm in the recent SENS-ILIAC trial [15]. Improvements in Rutherford classification and Ankle-Brachial Index (ABI) were comparable to previous studies. In BIONETIC-I, 93% of subjects experienced an improvement of ≥ 1 Rutherford class at 12 months, compared to 92.4% at 9 months in the VISIBILITY study. The average ABI at 12 months in BIONETIC-I was 0.93, versus 0.96 at 9 months in VISIBILITY.

In BIONETIC-I, 19.3% of lesions were in the external iliac artery (EIA). Unlike the SFA, which undergoes extreme kinking and compression through the adductor canal [28], the EIA remains relatively straight with limited mobility. BES is preferred for ostial lesions requiring precise placement and high radial support [29], while SES suits longer tortuous segments [30]. Our protocol selected BES for highly stenotic (minimum 70%, mean 85%) and calcified lesions (90% with calcification) because BES provides 5–10 times greater radial force than SES, essential for overcoming elastic recoil in rigid plaques [31]. While the ICE trial showed lower overall restenosis with SES (6.1% vs. 14.9%, *p* = 0.006), subgroup analysis revealed similar outcomes between BES and SES in heavily calcified lesions [19], supporting BES use for focal, severely calcified EIA lesions.

The BIONETIC-I trial showed that the Dynetic-35 stent had a consistent safety profile across all calcification levels, with similar MAE rates and clinical improvements even in severely calcified lesions. While 12-month outcomes align with prior studies, longer follow-up is needed. The results support bare metal stents as an effective, cost-efficient option for TASC A and B lesions, reserving covered stents for more complex cases.

## Conclusion

Despite treating complex iliac lesions, 30% severely calcified, the Dynetic-35 cobalt chromium stent demonstrated safety and efficacy over 12 months in the BIONETIC-I trial. Low rates of major adverse events (3.5%), revascularization (2.0%), and amputations (0.5%) were reported. Clinical improvements in ABI, Rutherford classification, and walking ability support its therapeutic value.

## Data Availability

The data underlying this study are available from BIOTRONIK AG, but are subject to restrictions and are not publicly accessible.

## Abbreviations

PAD: Peripheral artery disease
MAE: Major adverse events
cdTLR: Clinically driven target lesion revascularization
ABI: Ankle-Brachial Index
WIQ: Walking Impairment Questionnaire
CLTI: Chronic limb-threatening ischemia
CEC: Clinical Events Committee
PTA: Percutaneous transluminal angioplasty
BES: Balloon-expandable stent
TASC: TransAtlantic Inter-Society Consensus

## References

[1] Criqui MH, Aboyans V. Epidemiology of peripheral artery disease. Circ Res. 2015;116(9):1509–26. 25908725 10.1161/CIRCRESAHA.116.303849

[2] Javed IN, Hawkins BM. Aorto-iliac peripheral artery disease. Prog Cardiovasc Dis. 2021;65:9–14. 33631164 10.1016/j.pcad.2021.02.010

[3] Olin JW, Sealove BA. Peripheral artery disease: current insight into the disease and its diagnosis and management. Mayo Clin Proc. 2010;85(7):678–92. 20592174 10.4065/mcp.2010.0133 PMC2894725

[4] Libby P, Buring JE, Badimon L, Hansson GK, Deanfield J, Bittencourt MS, et al. Atherosclerosis. Nat Rev Dis Primers. 2019;5(1):56. 31420554 10.1038/s41572-019-0106-z

[5] Gerhard-Herman MD, Gornik HL, Barrett C, Barshes NR, Corriere MA, Drachman DE, et al. 2016 AHA/ACC guideline on the management of patients with lower extremity peripheral artery disease: a report of the American College of Cardiology/American Heart Association Task Force on Clinical Practice Guidelines. Circulation. 2017;135(12):e726–79. 27840333 10.1161/CIR.0000000000000471 PMC5477786

[6] Adam DJ, Bradbury AW. TASC II document on the management of peripheral arterial disease. Eur J Vasc Endovasc Surg. 2007;33(1):1–2. 17161778 10.1016/j.ejvs.2006.11.008

[7] Goode SD, Cleveland TJ, Gaines PA. Randomized clinical trial of stents versus angioplasty for the treatment of iliac artery occlusions (STAG trial). Br J Surg. 2013;100(9):1148–53. 23842828 10.1002/bjs.9197

[8] Bosch JL, Hunink MG. Meta-analysis of the results of percutaneous transluminal angioplasty and stent placement for aortoiliac occlusive disease. Radiology. 1997;204(1):87–96. 9205227 10.1148/radiology.204.1.9205227

[9] Aggarwal V, Waldo SW, Armstrong EJ. Endovascular revascularization for aortoiliac atherosclerotic disease. Vasc Health Risk Manag. 2016;12:117–27. 27099509 10.2147/VHRM.S98721 PMC4820232

[10] Milewski K, Zurakowski A, Pajak J, Pajak-Zielinska E, Liszka L, Buszman PP, et al. Comparison of thin-strut cobalt-chromium stents and stainless steel stents in a porcine model of neointimal hyperplasia. Med Sci Monit. 2010;16(1):BR40–4. 20037484

[11] Reekers JA, Vorwerk D, Rousseau H, Sapoval MR, Gaines PA, Stockx L, et al. Results of a European multicentre iliac stent trial with a flexible balloon expandable stent. Eur J Vasc Endovasc Surg. 2002;24(6):511–5. 12443746 10.1053/ejvs.2002.1775

[12] Rundback JH, Peeters P, George JC, Jaff MR, Faries PL. Results from the VISIBILITY iliac study: primary and cohort outcomes at 9 months. J Endovasc Ther. 2017;24(3):342–8. 28351204 10.1177/1526602817692960 PMC5438081

[13] Stockx L, Poncyljusz W, Krzanowski M, Schroe H, Allocco DJ, Dawkins KD. Express LD vascular stent in the treatment of iliac artery lesions: 24-month results from the MELODIE trial. J Endovasc Ther. 2010;17(5):633–41. 20939723 10.1583/09-2917MR.1

[14] Molnar RG, Gray WA. Sustained patency and clinical improvement following treatment of atherosclerotic iliac artery disease using the Assurant cobalt iliac balloon-expandable stent system. J Endovasc Ther. 2013;20(1):94–103. 23391088 10.1583/12-4010.1

[15] Choi WG, Rha SW, Choi BG. et al. Balloon-expandable cobalt chromium stent versus self-expandable nitinol stent for the Atherosclerotic Iliac Arterial Disease (SENS-ILIAC Trial) Trial: a randomized controlled trial. Heart Vessels. 2024;39:1060–7. 10.1007/s00380-024-02431-4. 10.1007/s00380-024-02431-4 38953938

[16] Torsello GF, Doerr B, Donas K, Berekoven B, Torsello GB, Beropoulis E. Treatment of iliac atherosclerotic lesions using the balloon-expandable dynamic bare metal stent: one-year outcomes of the BIODYNAMIC single-center retrospective analysis. Vascular. 2021;29(2):213–9. 32741310 10.1177/1708538120945422

[17] Laird JR, Loja M, Zeller T, Niazi KAK, Foster MT, Ansel G, et al. iCAST balloon-expandable covered stent for iliac artery lesions: 3-year results from the iCARUS multicenter study. J Vasc Interv Radiol. 2019;30(6):822-829.e4. 31031089 10.1016/j.jvir.2018.12.707

[18] Torsello GF, Stavroulakis K, Chlouverakis G, Torsello GB. Cobalt Chromium or Stainless Steel Balloon-Expandable Bare Metal Stents for Iliac Occlusive Disease? J Endovasc Ther. 2024;0(0). 10.1177/15266028241306068. 10.1177/15266028241306068 39711490

[19] Krankenberg H, Zeller T, Ingwersen M, Schmalstieg J, Gissler HM, Nikol S, et al. Self-expanding versus balloon-expandable stents for iliac artery occlusive disease: the randomized ICE trial. JACC Cardiovasc Interv. 2017;10(16):1694–704. 28838480 10.1016/j.jcin.2017.05.015

[20] Burket MW, Brodmann M, Metzger C, Tan K, Jaff MR. Twelve-month results of the nitinol Astron stent in iliac artery lesions. J Vasc Interv Radiol. 2016. 10.1016/j.jvir.2016.06.008. 27542591 10.1016/j.jvir.2016.06.008

[21] Clair DG, Adams J, Reen B, Feldman R, Starr J, Diaz-Cartelle J, et al. The EPIC nitinol stent system in the treatment of iliac artery lesions: one-year results from the ORION clinical trial. J Endovasc Ther. 2014;21(2):213–22. 24754280 10.1583/13-4560.1

[22] Hajibandeh S, Hajibandeh S, Antoniou SA, Torella F, Antoniou GA. Covered vs uncovered stents for aortoiliac and femoropopliteal arterial disease: a systematic review and meta-analysis. J Endovasc Ther. 2016;23(3):442–52. 27099281 10.1177/1526602816643834

[23] Mallory A, Giannopoulos S, Lee P, Kokkinidis DG, Armstrong EJ. Covered stents for endovascular treatment of aortoiliac occlusive disease: a systematic review and meta-analysis. Vasc Endovascular Surg. 2021;55(6):560–70. 33902342 10.1177/15385744211010381

[24] Mwipatayi BP, Sharma S, Daneshmand A, Thomas SD, Vijayan V, Altaf N, et al. Durability of the balloon-expandable covered versus bare-metal stents in the Covered versus Balloon Expandable Stent Trial (COBEST) for the treatment of aortoiliac occlusive disease. J Vasc Surg. 2016;64(1):83-94.e1. 27131926 10.1016/j.jvs.2016.02.064

[25] Malyar N, Behrendt C-A, Espinola-Klein C, Grözinger G, Lawall H, Nechwatal R, et al. S3-Leitlinie zur Diagnostik, Therapie und Nachsorge der peripheren arteriellen Verschlusskrankheit. Vasa. 2025;54(S113):1–119. 39996606 10.1024/0301-1526/a001171

[26] Laird JR, Zeller T, Holden A, Scheinert D, Moore E, Mendes R, et al. Balloon-expandable vascular covered stent in the treatment of iliac artery occlusive disease: 9-month results from the BOLSTER multicenter study. J Vasc Interv Radiol. 2019;30(6):836-844.e1. 30956077 10.1016/j.jvir.2018.12.031

[27] Bismuth J, Gray BH, Holden A, Metzger C, Panneton J. Pivotal study of a next-generation balloon-expandable stent-graft for treatment of iliac occlusive disease. J Endovasc Ther. 2017;24(5):629–37. 28697693 10.1177/1526602817720463

[28] Agrawal N, Eslami MH, Abou Ali AN, Reitz KM, Sridharan N. Adductor canal syndrome after lesser trochanter avulsion fracture in a 19-year-old. J Vasc Surg Cases Innov Tech. 2023;9(2):101098. 37101660 10.1016/j.jvscit.2023.101098 PMC10123372

[29] Canyiğit M, Beşler MS. Short-term outcomes of the iCover balloon-expandable covered stent for iliac artery lesions. Diagn Interv Radiol. 2025;31(1):52–7. 39155808 10.4274/dir.2024.242868 PMC11701692

[30] Squizzato F, Mosquera-Rey V, Zanabili Al-Sibbai A, Camblor Santervas LA, Pasqui E, Palasciano G, et al. Outcomes of self-expanding covered stents for the treatment of external iliac artery obstructive disease. Cardiovasc Intervent Radiol. 2023;46(5):579–87. 36826489 10.1007/s00270-023-03370-9 PMC10156894

[31] Brandt-Wunderlich C, Schmidt W, Thiesen A, Reinhardt F, Stiehm M, Schmitz K-P, et al. Test methods for evaluation of balloon expandable vascular stents – measurement of radial strength and stiffness. Curr Dir Biomed Eng. 2023;9(1):415–8.

